# Acupuncture treatment of a pregnant patient with Bell's palsy in the third trimester: Case report

**DOI:** 10.3389/fneur.2022.1088138

**Published:** 2023-01-04

**Authors:** Danchun Lan, Wenfei Deng, Kunze He, Qian Li, Xin Peng, Jinxiong Lao, Ziyong Li

**Affiliations:** ^1^Department of Acupuncture and Moxibustion, Foshan Hospital of Traditional Chinese Medicine, Foshan, China; ^2^The Eighth Clinical School of Medicine, Guangzhou University of Chinese Medicine, Foshan, China; ^3^Acupuncture and Rehabilitation Clinical School of Medicine, Guangzhou University of Chinese Medicine, Guangzhou, China

**Keywords:** acupuncture, Bell's palsy, pregnant patient, third trimester, case report

## Abstract

At present, the optimal treatment for Bell's palsy remains controversial, and the combination of corticosteroids and antiviral medication is usually recommended in the early stage. However, treatment is often delayed because the effects of these drugs on pregnant women and fetuses are still unclear. As a safe and effective complementary alternative therapy, acupuncture can alleviate Bell's palsy symptoms and improve the quality of life of the patient. Herein, we report the clinical presentation of a 27-year-old woman with Bell's palsy who was 26 weeks pregnant at the time of diagnosis. After five courses of treatment, the patient made a complete recovery.

## Background

Bell's palsy is the most common lower motor neuron facial palsy of the seventh cranial nerve, accounting for approximately 70% of all cases of facial nerve palsy. The annual incidence of Bell's palsy is 10–40 per 100,000 population. Pregnant women, especially in the third trimester and the first 2 weeks postpartum, are two to four times more likely to develop Bell's palsy than men and non-pregnant women. Moreover, the prognosis is more severe in pregnant women (1, 2).

The combination of corticosteroids and antiviral medication is usually recommended in the early stage of Bell's palsy. However, treatment is often delayed because the effects of these drugs on pregnant women and fetuses remain unknown (3). The use of steroids in pregnancy may affect the height, weight, and head circumference of newborns; lead to a cleft lip and palate; and worsen glucose control in patients with gestational diabetes (4–7). Therefore, effective drug therapy for Bell's palsy in pregnancy is limited.

As a vital part of traditional Chinese medicine, acupuncture is often used to treat Bell's palsy. While a number of studies (8–10) have certified the efficiency of acupuncture, one study showed that more research investigating the efficacy of acupuncture for Bell's palsy was necessary (11). There is also a lack of sufficient evidence to support acupuncture treatment for Bell's palsy in pregnant women. A 2010 study reviewed six small-sample RCTs that suggested the efficacy of acupuncture in treating Bell's palsy, but pregnant patients were not included in any of the six RCTs (12). Furthermore, another study revealed that acupuncture can improve Bell's palsy sequelae, but the study excluded pregnant patients (13).

**Figure 1:**
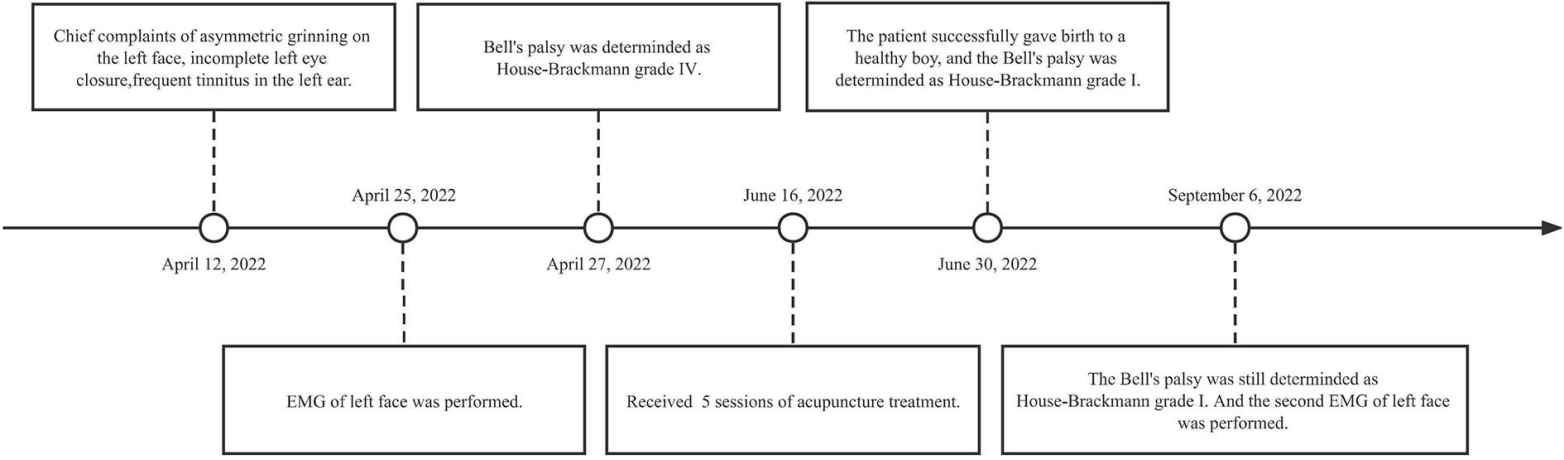
Timeline with relevant data from the episode of care.

Acupuncture is a safe and acceptable therapy for pregnant women with Bell's palsy (14). In this case, the safety of acupuncture is manifested not only in the half-needling technique that can control the depth of the subcutaneous tissue but also in the acupoints located in the four limbs and face, which will not harm pregnant women or fetuses. Additionally, no reinforcing or reducing methods are used. Therefore, pregnant women with Bell's palsy can benefit from acupuncture because they feel less pain and are more receptive to it, which will lead to better treatment compliance. In this case study, we report a case of Bell's palsy in a 26-week pregnant patient who was successfully treated with acupuncture (Figure 1).

## Case report

**Figure 2:**
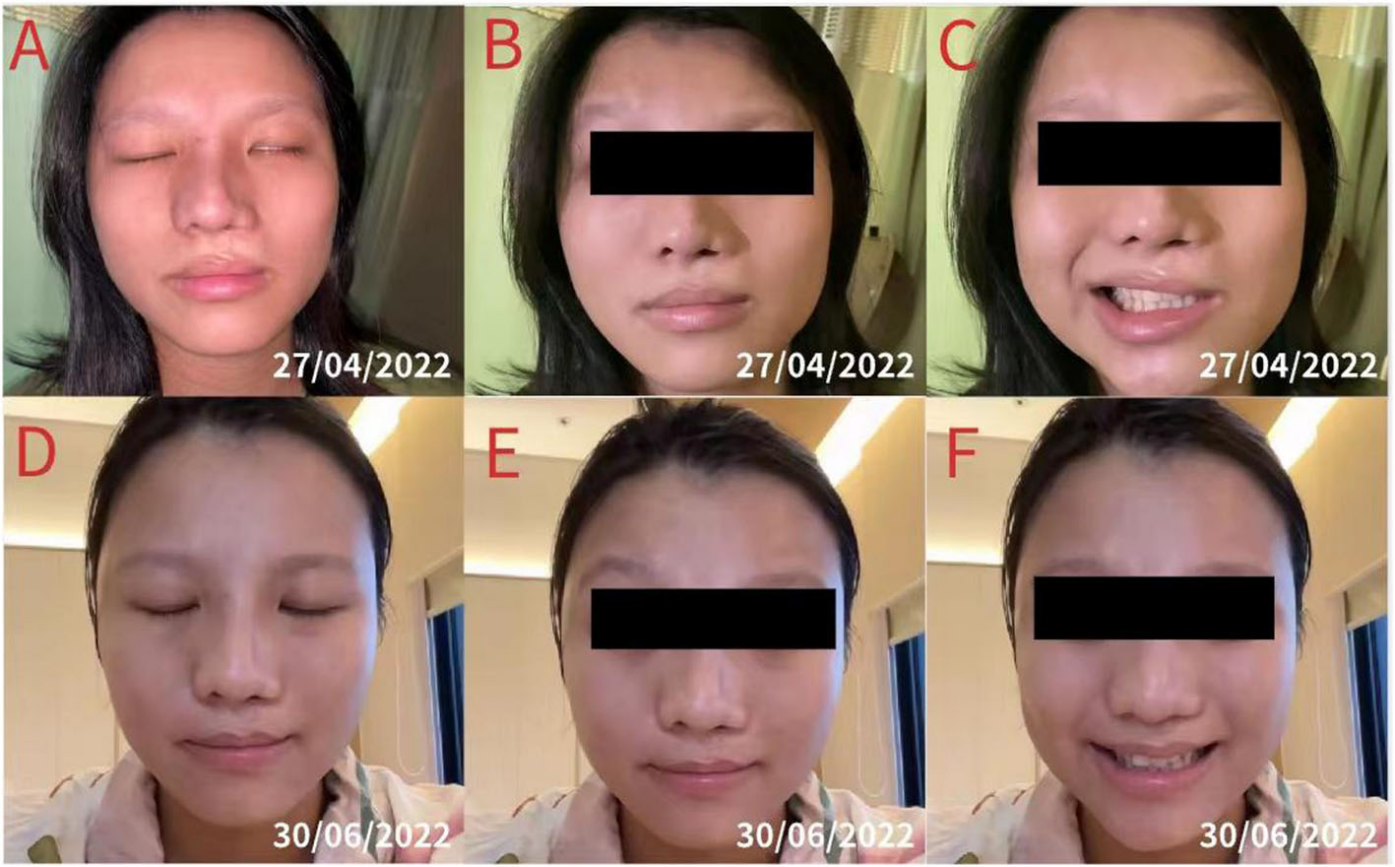
A patient with left-sided Bell's palsy. **(A)** Incomplete closure of the left eye and disappearance of creases on the forehead. **(B)** Inability to lift the left eyebrow. **(C)** Asymmetrical smiling and shallow left nasolabial fold. The patient received five sessions of acupuncture treatment. **(D)** Symmetrical closure of the eye. **(E)** Forceful raising of the left eyebrow and evident creases on the forehead. **(F)** Symmetrical smiling and an obvious left nasolabial fold.

On 12 April 2022, a 27-year-old woman had chief complaints of asymmetrical grinning on the left side of the face, incomplete left eye closure, frequent tinnitus in the left ear, and insomnia for the preceding 2 weeks, commencing at 26 weeks of gestation. The patient had no medical history of hypertension, diabetes, stroke, or Bell's palsy. On 25 April, electromyography (EMG) of the left side of the face showed that her left facial nerve was severely damaged, the zygomatic branch was completely damaged, and the left blink reflex could not be elicited. Moreover, the symptoms of the patient had not improved by then. Hence, she visited our clinic on 27 April to receive acupuncture treatment. Subsequent examination revealed an inability to complete left eye closure (Figure 2A) and lift the left eyebrow (Figure 2B); furthermore, we noted the disappearance of creases on her forehead. The patient was unable to shrug her left nose, puff out her cheeks, and smile symmetrically (Figure 2C). In addition, her left nasolabial fold became shallow. Given that the clinical manifestation of the patient was consistent with Bell's palsy, the diagnosis was confirmed (15).

Further assessment of facial nerve function revealed that the patient had House–Brackmann grade IV Bell's palsy. The House–Brackmann Facial Nerve Grading System (HBS) is the gold standard for evaluating facial nerve function and is used to evaluate the severity of facial paralysis. According to the HBS, nerve function is graded from I to VI, reflecting normal to total paralysis, in which grades I–III indicate mild facial paralysis and grades IV–VI indicate severe facial paralysis.

### Acupuncture treatment

**Figure 3:**
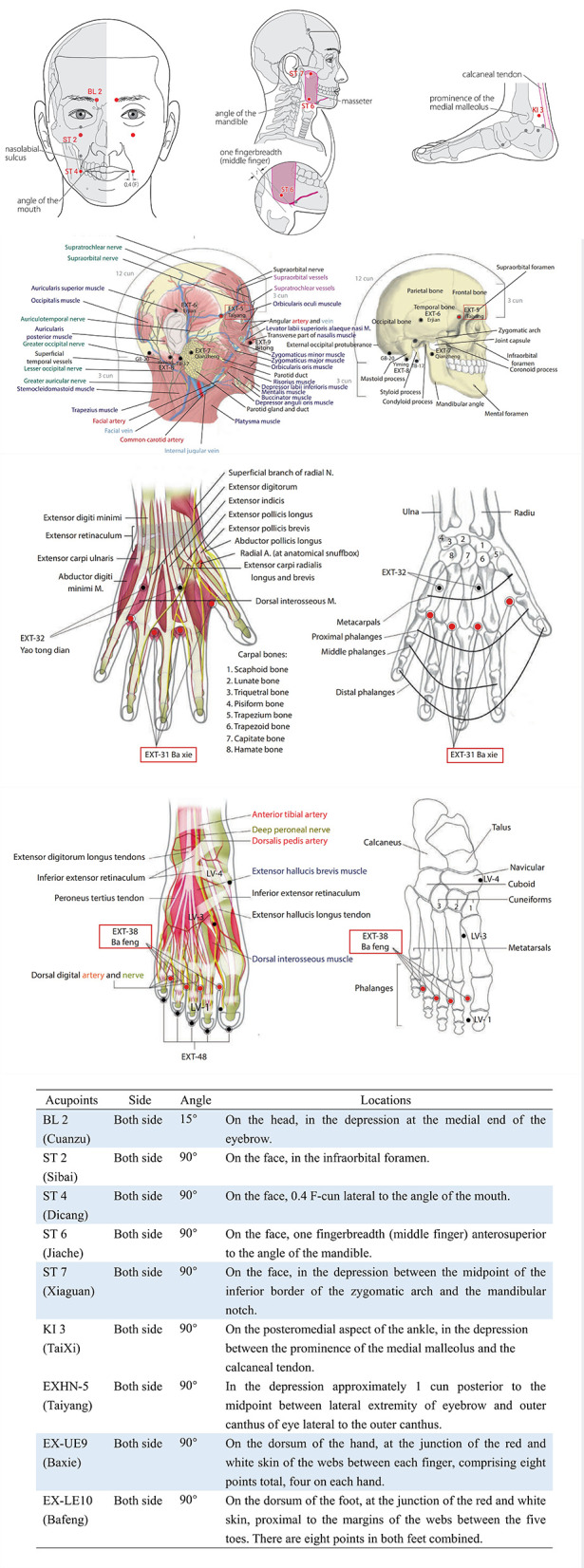
The locations of acupoints (16, 17).

The patient received five sessions of acupuncture treatment (one time a week for 5 consecutive weeks; all acupoints were retained for 30 min) without cointerventions. All sessions were performed by the same experienced acupuncturist registered in China. Each therapeutic session was consistent. A half-needling technique was used to stimulate the acupoints of the four limbs and the face, including BL 2 (Cuanzu), EXHN-5 (Taiyang), ST 2 (Sibai), ST 4 (Dicang), ST 6 (Jiache), ST7 (Xiaguan), EX-LE10 (Bafeng), EX-UE9 (Baxie), and KI 3 (TaiXi). The locations of the above acupoints are shown in Figure 3.

After selecting the acupoints and sanitizing the skin, needles (0.20 × 20 mm, Huanqiu Brand, China) were inserted at the acupoints. All needles were inserted to a depth of 5 mm, except for that inserted at the BL2 (Cuanzu) acupoint, which was pricked obliquely to the BL1 (Jingming) acupoint. Needles were inserted vertically at the other acupoints. All needles were stimulated to achieve De-Qi (characterized by soreness, heaviness, warmth, coolness, numbness, tingling, or distention around the acupoints) sensation and were retained for 30 min without the reinforcing and reducing methods. During treatment, she did not use any medication or other therapeutic aids except acupuncture.

### Clinical outcome

**Figure 4:**
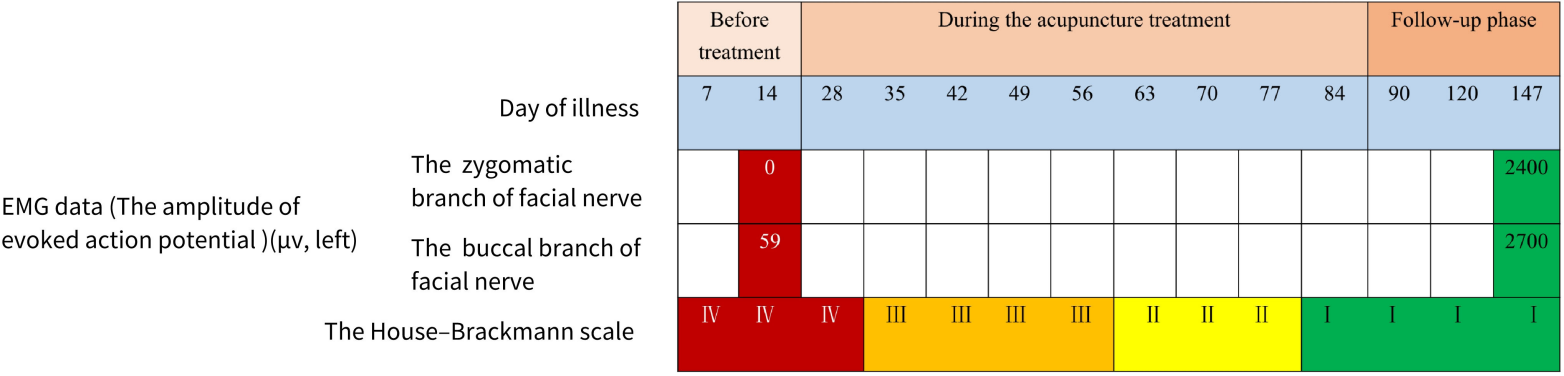
The EMG data and the House–Brackmann scale changed when the treatment progressed.

After two sessions of acupuncture treatment, the patient felt that her facial palsy had improved. She could lift her eyebrow slightly and almost close her eyes completely. Slight movement could be seen at the left corner of the mouth, and the asymmetry of her smile also slightly improved. The severity of facial paralysis was determined as HBS grade III (Figure 4). After four sessions of acupuncture treatment, her facial palsy continued to improve. The patient could completely lift her eyebrow and close her eyes. Creases on the left forehead were almost similar to those on the right forehead. In addition, the nasolabial fold was more evident than before, and the smile was slightly symmetrical. The severity of facial paralysis was determined as HBS grade II (Figure 4). The similarities between grade III and grade II are the weakness of facial muscles and asymmetrical movement, which indicates that Bell's palsy is not cured. The difference is the degree of facial dyskinesia; grade III takes the form of obvious, non-disfiguring weakness with complete eye closure, and grade II shows slight weakness on close inspection and a slight asymmetry of the smile. The change in the grade reflects the improvement of facial movement, especially in the orbicularis oris muscle.

Because the patient was approaching her due date of delivery, she discontinued acupuncture treatment. After 1 week, the patient successfully gave birth to a healthy baby boy, and the postpartum recovery of the patient was satisfactory. Using an online video examination, we found that the patient could symmetrically close her eyes (Figure 2D), raise her eyebrows (Figure 2E), shrug the left nose, puff out her cheeks, and smile (Figure 2F) 1 week after delivery. In addition, she had symmetrical folds around her nose and eyes. The severity of facial paralysis was determined to be HBS I (Figure 4), indicating that the patient had recovered.

The patient had retained the symmetrical facial expression during the 2-month follow-up (Figure 4). During the follow-up, the baby showed good health. The EMG result of the left face on 6 September 2022 indicated that the partially damaged facial nerve had improved. An action potential could be seen in the left zygomatic branch of the facial nerve, and the mixed nerve action potential (MNAP) of the left buccal branch of the facial nerve increased. The blink reflex showed that the latency of the left R1 was slightly longer than that of the right R1 (13.8 vs. 10.1 m/s). The latency of the left R2 and R2' were 42.2 and 41.6 m/s, respectively, which was significantly longer than those of the right R2 and R2', which were 26.4 and 24.8 m/s, respectively.

## Discussion

Our patient, who was 26 weeks pregnant, showed a significant decrease in HBS grade and an increase in the EMG data of the left facial nerve after five sessions of acupuncture.

### The etiology of Bell's palsy

The etiology of Bell's palsy remains unclear. Some studies suggested that the main cause of Bell's palsy is infection with reactivated viruses (18), such as the varicella-zoster virus (VZV) (19), herpes simplex virus type 1 (HSV-1) (20), human herpes virus 6 (21), and Usutu virus (22). HSV and VZV infections can be present throughout the life of the host (23). α-HVs enter the human body through the mucosa and localize in multiple ganglia of the neuroaxis *via* gene transcription, including in the autonomic and sensory ganglia of the head, the neck, and the cranium (24–27). In the case of immunodeficiency, HSV and VZV may get reactivated in the presence of circulating antibodies or an immunocompetent host (18). Some researchers proposed that immunosuppression in pregnant patients reduces the threshold for reactivation of the herpes virus in the geniculate ganglion, which increases the morbidity of Bell's palsy (28).

### Possible mechanisms of acupuncture on Bell's palsy in pregnancy

Acupuncture, which has a long history of being part of traditional China therapies, is often used to treat facial palsy and can effectively improve facial nerve dysfunction and reduce the severity of paralysis (10). In the United States, acupuncture has been considered one of the main therapies for complementary and alternative medicine (CAM) (29). The use of BL2, EXHN5, ST2, ST4, ST6, and ST7 on both sides of the face can ameliorate local blood circulation and promote the recovery of facial nerve function. The use of EX-LE10, EX-UE9, and KI3 of the four limbs can contribute to dispersing Qi. In traditional Chinese medicine, defensive Qi can help to resist external pathogens and protect the body. Defensive Qi is similar to the superficial tissue fluid of the body, and the flow of defensive Qi benefits the formation of adaptive immunity, which can improve overall immunity and prevent the reactivation of the herpes virus (30).

The use of the half-needling technique can mobilize the defensive Qi and elevate the acceptance and safety of acupuncture among patients.

The mechanisms of acupuncture on Bell's palsy are still not clear. Several studies suggest that, in patients with Bell's palsy, lower motor neuron paralysis reduces the movement feedback and breaks the connectivity of the cortical facial motor network, which leads to cerebral cortical reorganization and increases abnormal functional connectivity. The functional connectivity modulation induced by acupuncture may be beneficial to the recovery of diseases (31, 32). In addition, some studies proposed that acupuncture can promote the repair of facial nerve injury by altering the response of nerve fibers to electrical stimulation, accelerating the circulation of facial blood, relieving immunosuppression, and improving the expression of choline acetyltransferase (chAT) and neuritin (33–37).

### Treatment of Bell's palsy in pregnancy

Thus far, the optimal treatment method for Bell's palsy in pregnancy remains controversial. At present, corticosteroids are the main treatment agents for Bell's palsy. Patients with severe facial nerve palsy are recommended to undergo combination therapy with steroids and antiviral drugs (15). The French Society of ENT (SFORL) guidelines recommend that corticosteroids such as prednisolone or methylprednisolone should be ideally administered within 72 h of the onset of Bell's palsy. A study reported that corticosteroids should be administered for 3–10 days, which is conducive to the prognosis of Bell's palsy (38). Additionally, that study showed that the concentration of prednisolone in fetal serum is one-tenth of that in the maternal blood. Therefore, prednisolone can be used to treat Bell's palsy in the early stage of pregnancy (39). Nonetheless, the use of steroids in pregnancy is considered unsafe, and adverse drug reactions may unpredictably injure pregnant women and fetuses. Furthermore, there is very low certainty of evidence on surgery for the early management of Bell's palsy. Consequently, the efficacy and safety of surgery remain to be determined (40). In addition, physiotherapy, including external eyelid weighting and taping techniques for the cheek and the eye, has clinically provided benefits for patients during the rehabilitation period and can balance the movement of the face (41). Apart from those, depression, anxiety, and stressful life events are significant cases for pregnancy or postpartum. Therefore, early intervention is critical for pregnant women with Bell's palsy (42).

### Strengths and limitations

The clinical manifestation of the patient complied with the typical symptoms of Bell's palsy, which aided the prompt diagnosis. The clinical manifestation was paralysis of the unilateral facial muscle, resulting in the asymmetrical lifting of the eyebrows, forced closure of the eyes, shrugging nose, asymmetrical grinning, and disappearance of creases on the forehead and nasolabial fold. The disappearance of creases on the forehead could help differentiate it from central facial palsy.

Regarding diagnosis, a limitation of the study was the lack of magnetic resonance imaging (MRI). Hence, it was difficult to distinguish nuclear facial paralysis from lower sub-nuclear facial paralysis and rule out other diseases such as schwannoma, hemangioma, or a space-occupying lesion (43). One advantage of the evaluation used in this study lies in a variety of evaluation methods such as EMG and the HBS grading system to monitor the progress of facial nerve function over time. However, the follow-up period for this case was only 2 months, so it might be necessary to extend the follow-up period to evaluate the prognosis of Bell's palsy. Furthermore, the study showed that the main causes of neonatal Bell's palsy are congenital, developmental, and familial facial palsy (44, 45). Congenital palsy is related to perinatal trauma. Developmental palsy is related to an error in development (44). In addition, familial palsy occurs due to the inherited anatomical abnormality of the facial canal (46). According to the observation of the baby, perinatal trauma can be excluded. However, there needs to be a longer follow-up to observe the health of the baby to determine whether Bell's palsy can pass along to the baby and whether the acupuncture treatment can prevent it. Additionally, if the baby shows the symptoms of Bell's palsy, we will need to make a diagnosis by excluding the causes and performing an MRI. In addition, the EMG would need to be reevaluated after 6 months.

In this case study, the strengths of acupuncture treatment included the following: (1) All sessions were performed by experienced acupuncturists; (2) in every session, acupoints and acupuncture methods were consistent, which showed the normality and repeatability of the operation; (3) the low frequency of treatment (one time a week) could provide sufficient rest time for the patient; (4) the acupoints located in the face and four limbs were safer than those on the trunk; and (5) the Medical Classic of the Yellow Emperor emphasized that needles can be used to invigorate Zhengqi by light insertion, mild thrust, lift, and long retention. Therefore, all needles were retained without the reinforcing and reducing methods and electroacupuncture, which ensured that the patient was comfortable and helped them return to normal mood status. Although this case study showed that acupuncture may be a safe and effective treatment for Bell's palsy in pregnancy, high-quality, randomized controlled trials should still be conducted to validate our findings.

## Conclusion

Bell's palsy is an exclusionary diagnosis. The characteristic clinical manifestations are an important basis for diagnosis, and MRI is the best imaging modality to accurately exclude space-occupying lesions. EMG can evaluate the standard of facial nerve injury and determine the outcome of Bell's palsy. According to this case study, acupuncture can be used as soon as possible when the vital signs of pregnant women and fetuses are stable. The acupoints of the bladder meridian and stomach meridian on the face can ameliorate local blood circulation, and the combination with EX-LE10, EX-UE9, and KI3 can stimulate defensive Qi, which can warm the face, resist external pathogens, and improve the overall immunity. The half-needling technique is shallower in depth and safer and less uncomfortable, which can reduce the psychological burden of acupuncture. This case report demonstrated the feasibility, safety, and validity of acupuncture for treating Bell's palsy in pregnancy.

## Data availability statement

The original contributions presented in the study are included in the article/supplementary material, further inquiries can be directed to the corresponding authors.

## Ethics statement

The studies involving human participants were reviewed and approved by Ethics Committee of Foshan Hospital of Traditional Chinese Medicine (KY2022-255). The patients/participants provided their written informed consent to participate in this study. Written informed consent was obtained from the individual(s) for the publication of any potentially identifiable images or data included in this article.

## Author contributions

ZL performed the treatment and prepared the initial draft. KH, QL, and XP collected and analyzed the data. DL and WD were responsible for manuscript editing. JL was responsible for the review and overall supervision of the entire study. All the authors reviewed the final draft of the case report and accepted it for publication.
